# Supplementary material for: Revisiting the relative effectiveness of slaughterhouses in Ireland to detect tuberculosis lesions in cattle (2014–2018)
Source: PLoS One. 2022 Oct 7;17(10):e0275259. doi: 10.1371/journal.pone.0275259 (PMC9543943; doi:10.1371/journal.pone.0275259)
Supplement: S1 File — (ZIP) [file pone.0275259.s001.zip › Supporting Information/S1 Table.pdf]

S1 Table. Number of slaughtered animals, submission risk, and confirmation risk before exclusion of small factories, animal age below one year, and variables with missing values.

Factory	Number slaughtered	Number submitted	Submission (%)	Number confirmed	Confirmation (%)
S01	222,938	953	0.43	349	36.62
S02	87,900	273	0.31	134	49.08
S03	311,227	1031	0.33	492	47.72
S04	210,649	627	0.30	273	43.54
S05	259,293	735	0.28	239	32.52
S06	232,388	623	0.27	149	23.92
S07	28,891	76	0.26	24	31.58
S08	297,155	753	0.25	301	39.97
S09	320,123	801	0.25	366	45.69
S10	316,522	756	0.24	392	51.85
S11	387,298	888	0.23	301	33.90
S12	238,705	542	0.23	257	47.42
S13	224,596	496	0.22	190	38.31
S14	233,395	479	0.21	244	50.94
S15	225,332	433	0.19	103	23.79
S16	70,006	132	0.19	48	36.36
S17	229,641	429	0.19	116	27.04
S18	251,207	416	0.17	146	35.10
S19	17,616	29	0.16	9	31.03
S20	334,049	489	0.15	154	31.49
S21	230,142	310	0.13	162	52.26
S22	307,965	413	0.13	241	58.35
S23	106,777	141	0.13	61	43.26
S24	51,614	65	0.13	19	29.23
S25	283,899	350	0.12	137	39.14
S26	263,006	308	0.12	131	42.53
S27	145,728	153	0.10	95	62.09
S28	223,927	209	0.09	76	36.36
S29	368,812	338	0.09	175	51.78

S30	111,424	101	0.09	58	57.43
S31	13,427	2	0.01	0	0
S32*	2	0	0	0	0
S33*	332	0	0	0	0
S34*	1306	1	0.08	0	0
S35*	4562	10	0.07	5	50
**Total**	6,611,854	13,362	0.20	5447	40.76

*excluded factory
