# Supplementary material for: Revisiting the relative effectiveness of slaughterhouses in Ireland to detect tuberculosis lesions in cattle (2014–2018)
Source: PLoS One. 2022 Oct 7;17(10):e0275259. doi: 10.1371/journal.pone.0275259 (PMC9543943; doi:10.1371/journal.pone.0275259)
Supplement: S1 File — (ZIP) [file pone.0275259.s001.zip › Supporting Information/S2 Table.pdf]

S2 Table. The change of factories ranking per quartile between univariable and multivariable analysis.

Factory	Submission					Confirmation				
	Crude rank	Crude rank quartile	Adjusted rank	Adjusted rank quartile	Delta rank	Crude rank	Crude rank quartile	Adjusted rank	Adjusted rank quartile	Delta rank
S01	1	Q1	1	Q1	0	18	Q3	19	Q3	1
S02	2	Q1	6	Q1	4	10	Q2	11	Q2	1
S03	3	Q1	3	Q1	0	8	Q1	18	Q3	10
S04	4	Q1	7	Q1	3	12	Q2	15	Q2	3
S05	5	Q1	8	Q1	3	23	Q3	27	Q4	4
S06	6	Q1	2	Q1	4	30	Q4	29	Q4	1
S09	7	Q1	5	Q1	2	25	Q3	9	Q2	16
S10	8	Q1	4	Q1	4	15	Q2	13	Q2	2
S08	9	Q2	12	Q2	3	11	Q2	16	Q2	5
S07	10	Q2	9	Q2	1	6	Q1	8	Q1	2
S11	11	Q2	11	Q2	0	22	Q4	22	Q4	0
S12	12	Q2	10	Q2	2	9	Q2	7	Q1	2
S13	13	Q2	13	Q2	0	16	Q2	17	Q2	1
S14	14	Q2	19	Q3	5	7	Q1	14	Q3	7
S16	15	Q2	14	Q2	1	29	Q4	30	Q4	1
S15	16	Q2	15	Q2	1	21	Q3	20	Q3	1
S17	17	Q3	16	Q2	1	28	Q4	28	Q4	0
S19	18	Q3	18	Q3	0	20	Q3	24	Q4	4
S18	19	Q3	21	Q3	2	27	Q4	1	Q1	26
S23	20	Q3	20	Q3	0	26	Q3	21	Q3	5
S20	21	Q3	23	Q3	2	5	Q1	5	Q1	0
S22	22	Q3	25	Q4	3	2	Q1	6	Q1	4
S21	23	Q3	17	Q3	6	13	Q2	26	Q3	13
S25	24	Q3	26	Q4	2	24	Q4	25	Q3	1
S24	25	Q4	22	Q3	3	17	Q2	12	Q2	5
S26	26	Q4	24	Q3	2	14	Q3	10	Q2	4
S27	27	Q4	29	Q4	2	1	Q1	3	Q1	2
S29	28	Q4	27	Q4	1	19	Q3	23	Q3	4
S28	29	Q4	28	Q4	1	4	Q1	4	Q1	0
S30	30	Q4	30	Q4	0	3	Q1	2	Q1	1
S31	31	Q4	31	Q4	0	31	Q4	31	Q4	0
