# Supplementary material for: Revisiting the relative effectiveness of slaughterhouses in Ireland to detect tuberculosis lesions in cattle (2014–2018)
Source: PLoS One. 2022 Oct 7;17(10):e0275259. doi: 10.1371/journal.pone.0275259 (PMC9543943; doi:10.1371/journal.pone.0275259)
Supplement: Attachment — Submitted filename: Response to Reviewers.docx [file pone.0275259.s003.docx]

We appreciate very much the time and effort the reviewers have taken to read and comment on our manuscript. The comments and suggestion were very helpful to improve the paper and below you can find all our responses (in bold italics) with indications to where we have changed the manuscript. We hope that our answers to the queries are satisfactory and that the revised version is now acceptable for publication.

On behalf of all authors,

Rischi Robinson Male Here

(corresponding author)

Reviewer #1: The effort of the authors made in addressing the syntax and clarity of the manuscript were sound, and the methodology and model explanation are cleaer now. However, they were short in addressing many others concerning aspects concerning terminology (which could have been improved, but it wasn't) and, in particular, giving a broader context and depth to the results, and making the study a more interesting one.

***AU*: Thank you for your time and consideration. The terminology is in accordance with previous publications on the same topic both from the same group (Frankena, Olea-Popelka) as from other groups (Northern Ireland). Consistency indeed is important and we have the opinion to have done so.**

Reviewer #2: The revised version of the manuscript has satisfactorily addressed the points raised in the previous review. I have only a question and a couple of editorial suggestions
- I was a bit confused by one of the replies given to reviewer#1 regarding the purpose of multivariable models: this reply stated “in the univariable model only Factory_id was included as fixed effect (i.e. all factories) and herd_id as random effect”. From table 3 it seems obvious that in fact all available covariates were expressed in univariable models (this makes sense in my view). Based on the answer I assume that these univariable models also included a random effect (I think this also makes sense), and I assumed they did not include the factory (but based on the answer I am not so sure anymore). Perhaps this could be better clarified in lines 154-155 (version with track changes). A suggestion would be “The effect of each risk factor on the FL submission and confirmation risk was analysed with multilevel univariable logistic regression, followed by multilevel mixed effects logistic regression with…”, i.e., adding the multilevel to the univariable part to make evident that random effects were also included in the univariable models. Then, if all univariable models included the factory, perhaps state in that sentence this (since if the model includes e.g. factory and year does not fit the classical definition of a univariable regression model).

***AU*: Thank you for your comment and suggestion. We apologize and we understand your confusion. The reviewer is correct that all available covariates were expressed in univariable models as shown in Table 3. Also, we want to clarify that factory was not included in these univariable models. In the first rebuttal letter it was stated that herd was included as random effect in the univariable models; however the OR’s and CI’s in Table 3 originated from univariable models without that random effect. Therefore we corrected the univariable results presented in Table 3 and updated the text and figures where necessary, leading to very minor changes. Also we now explicitly mention in M&M that the univariable models included a random effect (line 152).**

Lines 234 and 260 (version in track changes): in the captions of figures 3 and 4, replace “referent” by “reference category”.

***AU*: Thank you for the suggestion. It has been replaced (line 230 and 255).**

**To the editor: we would like to update the funding disclosure to clarify the funder of the article processing costs. The updated funding disclosure is given below:**

**“The article processing costs associated with the publication of this study were provided by the Department of Agriculture, Food and Marine, Ireland. RRMH was sponsored by Indonesia Endowment Fund for Education scholarship (LPDP,** [**https://lpdp.kemenkeu.go.id**](https://eur03.safelinks.protection.outlook.com/?url=https%3A%2F%2Flpdp.kemenkeu.go.id%2F&data=05%7C01%7Cklaas.frankena%40wur.nl%7C47dba80540b9422daf4008da91c4445c%7C27d137e5761f4dc1af88d26430abb18f%7C0%7C0%7C637982569464321760%7CUnknown%7CTWFpbGZsb3d8eyJWIjoiMC4wLjAwMDAiLCJQIjoiV2luMzIiLCJBTiI6Ik1haWwiLCJXVCI6Mn0%3D%7C3000%7C%7C%7C&sdata=NGZps99h%2F4e%2F0uZ5SYTDdi%2BfMmFjjxHaLvFadV0c7SE%3D&reserved=0)**) from the Ministry of Finance, the Republic of Indonesia. The funder had no role in study design, data collection, and analysis, decision to publish, or preparation of the manuscript”.**
